# Community Water Fluoridation Levels To Promote Effectiveness and Safety in Oral Health —  United States, 2016–2021

**DOI:** 10.15585/mmwr.mm7222a1

**Published:** 2023-06-02

**Authors:** Theresa J. Boehmer, Srdjan Lesaja, Lorena Espinoza, Chandresh N. Ladva

**Affiliations:** ^1^Division of Oral Health, National Center for Chronic Disease Prevention and Health Promotion, CDC; ^2^DB Consulting Group, Inc., Bethesda, Maryland.

Drinking water fluoridated at the level recommended by the U.S. Public Health Service (USPHS) reduces dental caries (cavities) by approximately 25% in children and adults (*1*). USPHS recommends fluoride levels to achieve oral health benefits and minimize risks associated with excess fluoride exposure. To provide the benefits of community water fluoridation, water systems should target a level of 0.7 mg/L and maintain levels ≥0.6 mg/L (*2*). The Environmental Protection Agency (EPA) sets a safety standard at 2.0 mg/L to prevent mild or moderate dental fluorosis, a condition that causes changes in the appearance of tooth enamel caused by hypermineralization resulting from excess fluoride intake during tooth-forming years (i.e., before age 8 years). During 2016–2021, fluoride measurements for 16.3% of population-weighted monthly fluoride measurements (person-months) reported by community water systems to CDC’s Water Fluoridation Reporting System (WFRS) were <0.6 mg/L; only 0.01% of person-months exceeded 2.0 mg/L. More than 80% of population-weighted fluoride measurements from community water systems reporting to WFRS were above 0.6 mg/L. Although 0.7 mg/L is the recommended optimal level, ≥0.6 mg/L is still effective for the prevention of caries. A total of 4,080 community water systems safely fluoridated water 99.99% of the time with levels below the secondary safety standard of 2.0 mg/L. Water systems are encouraged to work with their state programs to report their fluoride data into WFRS and meet USPHS recommendations to provide the full benefit of fluoridation for caries prevention.

Monthly data from WFRS during 2016–2021 were analyzed for water systems that added fluoride (adjusting systems); these systems provide monthly average fluoride levels in mg/L. These monthly average fluoride levels were compared for two goals: prevention and safety. For prevention, reported levels were compared with 0.7 mg/L, the USPHS-recommended optimal fluoride level for preventing caries (*3*). For safety (i.e., to minimize potential fluorosis)* (*4*), reported fluoride levels were compared with the EPA’s secondary maximum contaminant level (SMCL), of 2.0 mg/L. All analyses were conducted using SAS (version 9.4; SAS Institute) and R (version 4.1.3; The R Foundation). This activity was reviewed by CDC and was conducted consistent with applicable federal law and CDC policy.^†^

Water system populations were obtained from WFRS for each year during 2016–2021 (*5*). These populations are updated periodically by the states directly to WFRS or annually by CDC from EPA’s State Drinking Water Information System. Population-weighted monthly fluoride levels (person-months) were calculated by multiplying each average monthly fluoride level by the size of the population served by each water system.

Data are typically reported to WFRS on a monthly, quarterly, or yearly basis. Participation across states varies based on fluoride-reporting requirements, drinking water or oral health program staffing limitations, and fluoridation program funding status. Among approximately 54,000 water systems in WFRS, a total of 5,888 adjust fluoride levels and serve a population of more than 200 million persons (145 million directly and an additional 55 million through water systems that purchase fluoridated water from adjusted water systems). Among the systems in WFRS, a total of 4,080, serving a population of 124,616,896, provided at least 1 month of data during the study period. Among 7,936,442,898 person-months during 2016–2021, only 796,283 (0.01%) exceeded the SMCL^§^; 16.3% were below 0.6 mg/L, and 83.7% of person-months operated between 0.6 mg/L and 2.0 mg/L with the largest peak in data at the 0.7 mg/L target (Figure).

**FIGURE:**
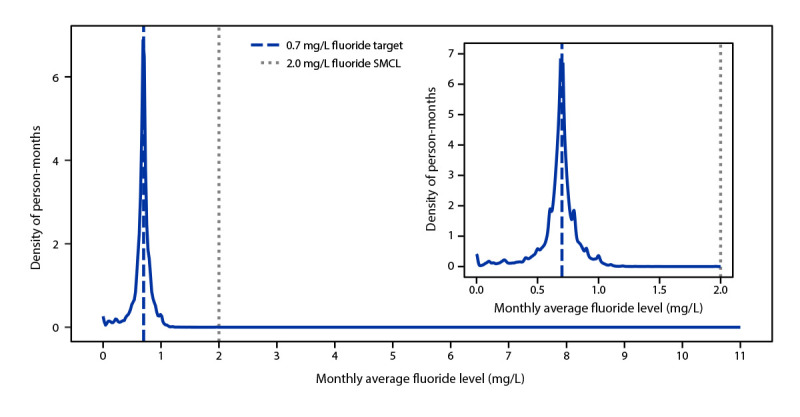
Density estimation of population-weighted monthly average fluoride levels — United States, 2016–2021 **Abbreviation:** SMCL = secondary maximum contamination level.

## Discussion

In this examination of the performance of U.S. water systems reporting fluoride levels from the perspectives of preventing caries and supporting established safety standards, the most common person-month fluoride level was the USPHS-recommended level of 0.7 mg/L and fluoride levels rarely exceeded the SMCL (0.01%). SMCL exceedances should be minimized to reduce dental fluorosis. Dental caries are one of the most common preventable chronic diseases among U.S. children: approximately one in four children living below the federal poverty level experiences untreated caries (*6*). Optimal levels of water fluoridation prevent caries by providing frequent and consistent contact with low levels of fluoride, ultimately reducing tooth decay by 25% in children and adults (*7*). Water systems that consistently and optimally fluoridate support the reduction of tooth decay. Suboptimal water systems in which fluoride concentrations are <0.6 mg/L are both ineffective in using resources and in supporting the oral health of their communities. Optimal fluoridation can be maintained with routine maintenance and monitoring, which provide protection from equipment malfunction, disruptions in fluoride supply, and periodic system shutdowns.^¶^

Water fluoridation promotes health equity through its proven effects on decreasing caries, reducing costs to families, and being readily available at the tap. In light of these benefits, Healthy People 2030, an ongoing initiative to improve population health, set the objective to increase the proportion of U.S. residents served by optimally fluoridated water systems to 77.1% from 73.0% in 2018 (*8*). Currently, programs nationwide receive a net savings of $6.5 billion per year by averting direct dental treatment costs (tooth restorations and extractions) and indirect costs (follow-up treatment and losses of productivity) (*9*). After community water fluoridation was discontinued in Juneau, Alaska, for example, a higher number of caries-related procedures among persons aged <18 years was documented, particularly in persons born after cessation of fluoridation, highlighting the long-term oral health benefits of supporting access to fluoridated water (*10*).

The findings in this report are subject to at least two limitations. First, CDC relies on state oral health and drinking water programs to report operational information; 31% of adjusting systems (5,888) did not report any fluoride levels during 2016–2021. Second, population values for all water systems are obtained from EPA’s State Drinking Water Information System federal database at the state’s discretion; however, additions and deletions of water systems and associated fluoridation status must be received from the state programs. As a result, counts of water system and information might differ from other publicly available community water system databases. Reporting in WFRS might be increased by improving data sharing between state drinking water and oral health programs, especially in states where water system data are entered into WFRS by the oral health program. Methods to increase reporting can include creating a data-sharing memorandum of understanding between the two programs and implementing a state policy that requires water systems to conduct monthly recording and reporting to the state.

Thousands of fluoride-adjusting community water systems reach approximately 200 million persons in the United States. To promote receipt of the full benefits of community water fluoridation, water systems must manage resources to meet the established 0.7 mg/L target consistently, especially those serving communities where fluoride measurements were <0.6 mg/L. CDC carefully and continuously monitors emerging research about the benefits and risks of fluoride exposure so that recommendations are evidence-based. CDC continues to emphasize the importance of community water fluoridation at the recommended level of 0.7 mg/L as the cornerstone of dental caries prevention in the United States.** Water systems are encouraged to work with their state programs to report their fluoride data into WFRS and meet USPHS recommendations to provide the full benefit of fluoride in caries prevention. Maintaining and improving access to optimally fluoridated water remains a vital, safe, and successful method for reducing dental caries and their associated costs for communities and families.

**Summary**

**What is already known about this topic?**

Community water fluoridation delivers cavity-preventing fluoride to everyone with access. The U.S. government sets optimal fluoridation at 0.7 mg/L and a safety standard at 2.0 mg/L.

**What is being added by this report?**

During 2016–2021, a total of 4,080 community water systems safely fluoridated water 99.99% of the time, with levels below the secondary safety standard of 2.0 mg/L. However, 16.3% of nearly 8 billion population-weighted monthly fluoride measurements were <0.6 mg/L, placing the prevention of cavities in jeopardy.

**What are the implications for public health practice?**

Water system managers are encouraged to work with their state programs to report fluoride data to CDC and meet U.S. Public Health Service recommendations to provide the full benefit of cavity prevention through water fluoridation.

## Footnotes

* EPA also sets a maximum contaminant level (MCL) of 4.0 mg/L to prevent bone disease and mottling of teeth from fluorosis.

† 5 C.F.R. part 46, 21 C.F.R. part 56; 42 U.S.C. Sect. 241(d); 5 U.S.C. Sect. 552a; 44 U.S.C. Sect. 3501 et seq.

§ Fluoride levels rarely exceeded the MCL (0.002%).

¶
https://www.cdc.gov/mmwr/PDF/rr/rr4413.pdf

**
https://www.cdc.gov/fluoridation/guidelines/cdc-statement-on-community-water-fluoridation.html
